# Supplementary material for: Are religious patients less afraid of surgery? A cross-sectional study on the relationship between dimensions of religiousness and surgical fear
Source: PLoS One. 2023 Jul 13;18(7):e0287451. doi: 10.1371/journal.pone.0287451 (PMC10343077; doi:10.1371/journal.pone.0287451)
Supplement: Attachment — Submitted filename: Title suggest to change.docx [file pone.0287451.s002.docx]

Title suggest to change

**Relationship between preoperative surgical fear and religiosity dimensions**

**Line 53	cross sectional study among surgical clinics in one hospital?**

**Line 54	setting identify the name of the** tertiary facility **/hospital**

**Line 78 key words :- no need to repeat preoperative**

94 could you mention the study significance prevalence of fear among Croatian patients

Line 116	needs to be dissected, taking into account the different potential effects ( need rephrases )

**161	elective surgery you mean ( regardless the type of surgery ) ?**
