# Supplementary material for: Are religious patients less afraid of surgery? A cross-sectional study on the relationship between dimensions of religiousness and surgical fear
Source: PLoS One. 2023 Jul 13;18(7):e0287451. doi: 10.1371/journal.pone.0287451 (PMC10343077; doi:10.1371/journal.pone.0287451)
Supplement: Attachment — Submitted filename: Response.docx [file pone.0287451.s003.docx]

Response to Reviewers

Dear Reviewers,

thank you very much for your constructive review and all the compliments. I hope to have incorporated all your commentaries into the new version of the manuscript.

Below my replies to the reviewers:

- as suggested, I have changed the manuscript title by adding the aspect of dimensions.
- I have specified the details in the Design and Setting subsection of the Abstract as instructed.
- I have crossed out the repeated term in the Key words.
- I cannot mention the prevalence of fear among Croatian patients, because up-to-date only one study has been completed by our group. But, this study did not assess the presence or absence of surgical fear but the level of surgical fear, so we cannot present data on how many patients in Croatia indicate surgical fear, respectively indicate no surgical fear at all.
- I have rephrased the sentence in Line 116, now 117 and hope that this formulation is more comprehensible.
- in line 161 I have written that all patients admitted for any elective surgery, regardless of type of surgery were potential candidates.
- I have added the reference to our previous study on surgical fear where we have validated the Croatian version of the SFQ, which has been accepted for publication, but has not yet been published.
- I have added passages in the Results section where I have commented on the results of Table 1 and extended my commentaries on Table 2.
- Table 4 was broken up into two tables to render a clearer graphic representation of the respective results.
- A thorough check for grammatical error in the whole manuscript, not only line 392 was performed.
- The suspected missing part in the Methods section was a place-holder and deleted.
- The reviewers suggested to disclose more information on the patient population who reported no religious behaviors. This was a thought of ours, but since the manuscript focuses on religious patients and their relationship with surgical fear, we did not include data on this specific topic. Additionally, we realized that data analysis on this subject would yield only information about sociodemographic factors regarding this subgroup, not novel insight into the relationship between religiousness and surgical fear.
- All references have been checked for the APA style.

Kind regards,

Andrija
